# Senescent skeletal cells cross-talk with synovial cells plays a key role in the pathogenesis of osteoarthritis

**DOI:** 10.1186/s13075-022-02747-4

**Published:** 2022-02-28

**Authors:** Chong-Jie Wu, Ri-Xu Liu, Song-Wei Huan, Wang Tang, Yu-Kai Zeng, Jun-Cheng Zhang, Jie Yang, Zhen-Yan Li, Ying Zhou, Zhen-Gang Zha, Huan-Tian Zhang, Ning Liu

**Affiliations:** 1 Department of Bone and Joint Surgery, the First Affiliated Hospital, Jinan University, Guangzhou, 510630 Guangdong China; 2 Institute of Orthopedic Diseases & The Bone and Joint Disease institute of Guangdong-Hong Kong-Macao Greater Bay Area, Jinan University, Guangzhou, 510630 China

**Keywords:** Osteoarthritis, Skeletal cells, Cellular senescence, SASP, Targeted therapies, Animal models

## Abstract

Osteoarthritis (OA) has been recognized as an age-related degenerative disease commonly seen in the elderly that affects the whole “organ” including cartilage, subchondral bone, synovium, and muscles. An increasing number of studies have suggested that the accumulation of senescent cells triggering by various stresses in the local joint contributes to the pathogenesis of age-related diseases including OA. In this review, we mainly focus on the role of the senescent skeletal cells (chondrocytes, osteoblasts, osteoclasts, osteocyte, and muscle cells) in initiating the development and progression of OA alone or through cross-talk with the macrophages/synovial cells. Accordingly, we summarize the current OA-targeted therapies based on the abovementioned theory, e.g., by eliminating senescent skeletal cells and/or inhibiting the senescence-associated secretory phenotype (SASP) that drives senescence. Furthermore, the existing animal models for the study of OA from the perspective of senescence are highlighted to fill the gap between basic research and clinical applications. Overall, in this review, we systematically assess the current understanding of cellular senescence in OA, which in turn might shed light on the stratified OA treatments.

## Introduction

Osteoarthritis (OA) is the most common age-related disease that affects the elderly [1]. Currently, it is estimated to be the fourth leading cause of pain and physical disability (deformity and limited range of motion) [2]. A great number of patients with late-stage OA have to undergo surgical interventions [3, 4]; thus, OA has a significant impact on the individuals, health-care systems, and society [5, 6]. A better understanding of the precise mechanisms that drive OA progression will shed light on the stratified treatment of OA.

Cellular senescence is an important process induced by various types of stresses that leads to irreversible proliferative cell cycle arrest and distinctive cellular phenotypic alteration [6–9]. Recently, several studies have highlighted the crucial role of senescent skeletal cells in the development and progression of OA [10, 11]. Additionally, the cross-talk of synovial cells in a joint with various senescent musculoskeletal cells may facilitate the pathogenesis of OA by amplifying the overlapping inflammatory response and/or by triggering the senescence-associated secretory phenotype (SASP) [12]. Accordingly, therapies aiming to eliminate the senescent cells are investigated in a series of OA models [13]. These findings have deepened our understanding of mechanisms of OA from the perspective of cellular senescence and thereby suggesting novel strategies for stratified OA therapy.

### Key stimuli for cellular senescence and OA

Cellular senescence was first described by Hayflick and Moorhead in 1961, in which two main types of cellular senescence, e.g., replicative senescence and premature senescence, were included [14]. Replicative senescence refers to a state of reduced replicative capacity causing by telomere shortening, while premature senescence which defined as cell cycle arrest is induced by oxidative stress and DNA damage [15]. Typical features of this type of cellular senescence included increasing the expression of p21, p16, and p53, and elevated the senescence-associated-β-galactosidase (SA-β-Gal) activity and high levels of reactive oxygen species (ROS) [16]. The liberation of p16 suppression caused early cell senescence [17], while p53, p21 (CIP1) and p15 (INK4b) increased in pre-senescent cells [18]. On the other hand, the increase of Bcl-XL in pre-senescence cells enhanced their own anti-apoptotic ability, which made the cells progress to senescence rather than apoptosis [19]. In addition, the metabolic activities and secretion levels of the SASP and the proinflammatory cytokine in the senescent cells were demonstrated to be altered [20]. In a recent study, it was found that the combination of mitogenic stimulation and DNA damage induced chondrocyte senescence and increase the production of senescence marker: SA-β-Gal, p16, and γH2AX, etc. [21].

#### Telomeres

Telomeres are nucleoprotein structures at the ends of linear chromosomes that ensure genome stability by protecting DNA extremities from fusion and degradation [22]. In the process of cell replication, telomeres gradually shorten and lose the function of protecting DNA stability. As a result, DNA repair machinery is initiated during the DNA damage response which leads to aging [23]. Chondrocytes in osteoarthritic cartilage may undergo a replicative senescence with associated phenotypic changes leading to the development of OA. Through a comparison of clinical specimens in a group of 15 patients with hip OA, 30 with knee OA, and 11 controls without joint disease, it has been found that the telomeres of OA patients were shorter than unaffected chondrocytes of controls [24]. In addition, the function of telomeres in protecting DNA is also related to telomerase. Cells lacking telomerase triggers the DNA damage response, which induces early onset of senescence thereby promoting the progression of osteoarthritis [25, 26].

In addition, oxidative stress and inflammation have been considered as important factors that accelerate the aging process by shortening telomeres and/or causing telomere abrasion [27, 28]. This conclusion has been supported by a recent study showing that the relative telomere length (RTL) of leukocytes in knee OA is shorter than in age-matched healthy controls and that a negative correlation between oxidative stress, inflammatory factor levels, and leukocyte RTL is found in the knee joints of OA patients [29]. It is obvious from these previous findings that telomere length (TL) interventions can significantly slow the aging process, which highlighted an avenue for treating age-related diseases such as OA.

#### Oxidative stress

Oxidative stress is widely accepted as a major inducer of DNA damage and cellular senescence [30]. Mechanistically, it is demonstrated to induce cell senescence by upregulating the expression of p53 and p21, as well as activating the p38 mitogen-activated protein kinase (MAPK) and phosphatidylinositol 3 kinase (PI3K)/protein kinase B (PKB) signaling pathways [31–33]. Previous studies have shown that the progression of OA is significantly associated with oxidative stress and ROS levels [34, 35]. Oxidative stress is regulated by MAPK pathway members such as Jun N-terminal kinases (JNKs) and p38 [31]. It has been reported that the deletion of JNK1 and JNK2 in mice leads to more obvious cellular senescence in the cartilage and synovium than is observed in wild-type mice, thereby exhibiting a more severe OA phenotype [36]. This suggests that JNK plays an important role in the progression of age-induced OA. In addition, studies have found that intracellular ROS levels, especially superoxide anion levels, are elevated in posttraumatic OA mouse model [20]. The accumulation of superoxide anions is associated with a downregulation of its degrading enzyme, mitochondrial superoxide dismutase 2 (SOD2) [37]. This association has also been demonstrated in patients with OA. The expression of SOD is downregulated and the production of intracellular superoxide is increased [38].

On the other hand, overproduction of ROS might also increase oxidative stress, and the latter plays a crucial role in the destruction of homeostasis in articular cartilage by suppressing autophagy, which together aggravates the severity of OA [39, 40]. Consistently, oxidative stress, which is related to an increase in ROS and a decrease in antioxidant levels, is considered to be an important factor in the occurrence and development of OA. This evidence was obtained from a recent study demonstrating that the ROS scavenger vitamin C prevents chondrocyte senescence in PTOA rat models [41], indicating that modulation of oxidative stress might be beneficial for cartilage by reducing ROS. Therefore, the imbalance between ROS production and the antioxidant capacities of cells is now considered a potential factor in the development of OA (Table 1).

**Table 1:** Telomeres and oxidative stress contribute to cellular senescence also related to OA

Factors	Correlation with senescence	Refs	Correlation with OA	Refs
Telomeres	DNA repair mechanisms recognized telomere shortening triggers the DNA damage response leading to senescence	[23]	Negative associations of oxidative stress and inflammatory factors levels with RTL in the knee joints of OA patients	[29]
	Cells lacking telomerase trigger the DNA damage response leading to senescence	[25]	Shorter TL in OA patients	[24]
	Oxidative stress accelerates cellular senescence by shortening telomeres	[27]		
	Inflammation can lead to telomere abrasion inducing cellular senescence	[28]		
Oxidative stress	ROS accumulation, cell cycle arrest and cellular senescence accompanied by upregulation of p53 and p21 proteins in cells	[31]	Increased levels of intracellular ROS disrupt cartilage homeostasis and lead to cartilage damage in OA	[39]
	Cellular senescence is largely dependent on ROS	[34, 35]	Intracellular ROS level were elevated in posttraumatic OA	[20]
	ROS induced premature senescence by PI3K/Akt/mTOR pathway	[33]	The production of intracellular superoxide is increased in OA patients	[38]

*OA* osteoarthritis, *ROS* reactive oxygen species, *TL* telomere length, *RTL* relative telomere length

### Key signaling pathways for senescent cells and OA

#### Signaling pathways associated with senescence

The essential role of p53, p16, and p21 in cellular senescence has been extensively investigated; basically, they can be categorized into two major signaling pathways: the p53/p21 and the Rb/p16. Previous studies have revealed a higher expression of p16 in the articular chondrocytes of elderly mice and humans [42]. Remarkably, the selective elimination of p16 in p16 overexpressing cells can prolong the life span of mice [43].

Many stress stimuli can activate different signaling pathways and eventually cause cell cycle arrest via the p53/p21 and/or the Rb/p16 pathways. Although these two pathways share several common regulatory factors, p16/pRB pathway-induced cell growth arrest has been considered irreversible, while p53/p21 pathway-induced cell growth arrest is reversible [44]. A recent study has confirmed that activation of the Wnt/β-catenin signaling pathway plays a role in the development of OA by downregulating the sirtuin 1(SIRT1) expression, while upregulating the expression of acetylated p53 and thus promoting the senescence of chondrocytes [32]. Omentin-1 is a newly discovered anti-inflammatory adipokine, and the expression of omentin-1 has been shown to reduce the expression of p21 and p53 acetylation by suppressing the SIRT1 reduction [45]. This study offers a possible treatment option for OA by the utilization of omentin-1.

#### Signaling pathways relevant to the maintenance of senescent cells

**Fig. 1:**
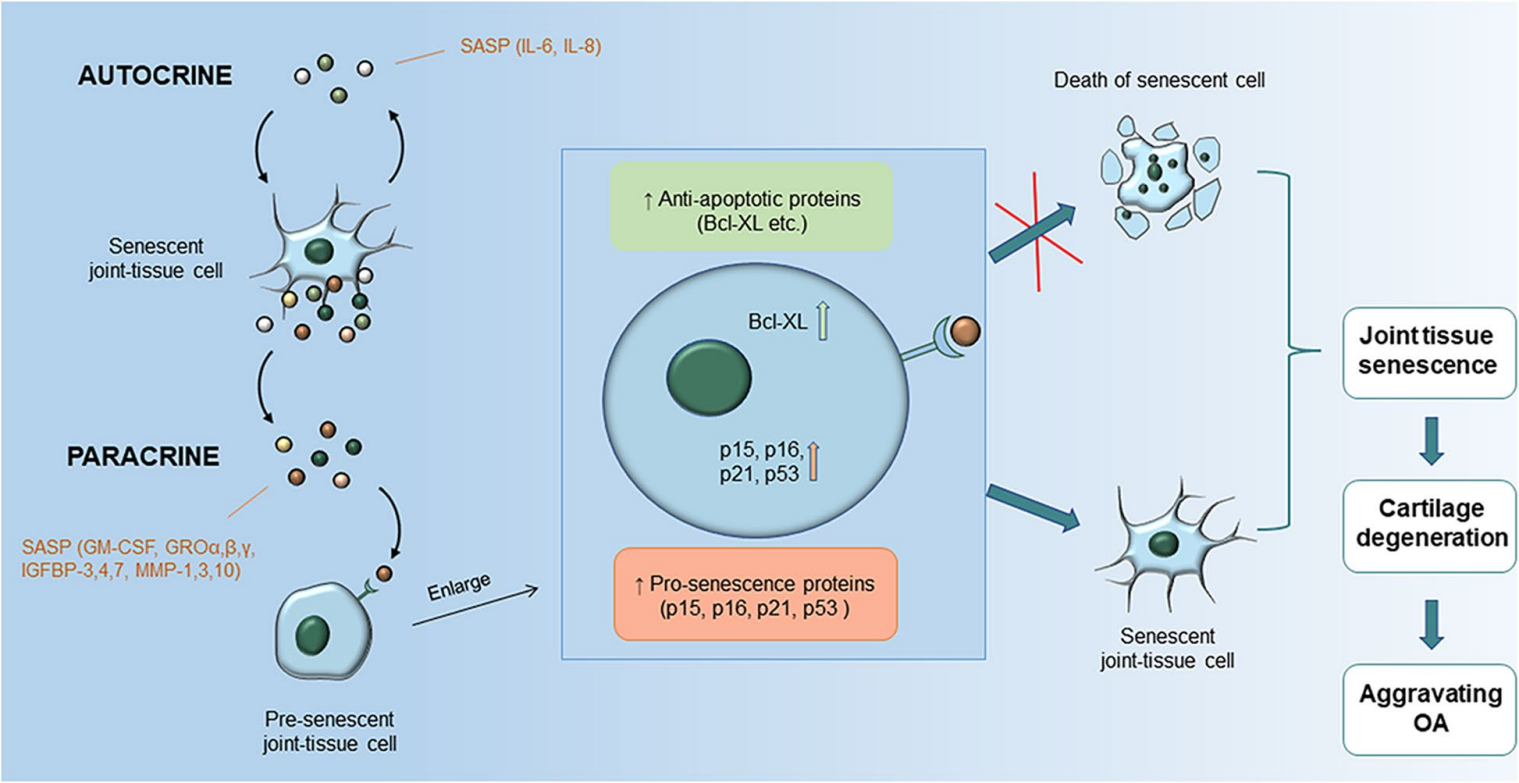
SASP secreted by senescent cells accelerates senescence of surrounding cells. Cytokines such as IL-6 and IL-8 in SASP can act on senescent cells themselves in the form of autocrine, while many other SASP molecules are transmitted from cell to cell in a paracrine form to cause senescence in the neighboring cells, thus exacerbating the pro-senescence effect. p53, p21, p15, and p16 are increased in the pre-senescent cells. On the other hand, the increase of Bcl-XL in the pre-senescence cells enhances their anti-apoptotic ability, which renders the cells undergo senescence instead of apoptosis. Senescent cells within the joint accelerate the progression of OA. IL, Interleukin; SASP, senescence-associated secretory phenotype; GM-CSF, granulocyte macrophage-colony stimulating factor; GRO, growth-regulated oncogene; IGFBP, insulin-like growth factor-binding protein; MMP, matrix metalloproteinase; OA, osteoarthritis

Senescent cells not only exhibit the characteristics of growth stagnation, but also favor the SASP, through which several extracellular proteases, inflammatory cytokines, growth factors, and chemokines such as granulocyte macrophage-colony stimulating factor (GM-CSF), growth-regulated oncogene (GRO)α, β, γ, insulin-like growth factor-binding protein (IGFBP)-3, IGFBP-4, IGFBP-7, interleukin (IL)-1α, IL-6, IL-7, and IL-8 are secreted [46, 47]. The components of the SASP are highly cell-type-dependent and vary according to the inducer of senescence, the duration of senescence, and the surrounding microenvironment [48]. Studies have demonstrated that there is an overlap of the SASP and the secreted proinflammatory factors in skeletal cells [49–51]. The SASP molecules can exacerbate and spread pro-senescence effects by acting on senescent cells as well as neighboring cells through either autocrine and/or paracrine manner. Factors such as IL-6 and IL-8 might reinforce the senescent state in an autocrine manner in senescent cells, while many other SASP factors cause senescence in neighboring cells through paracrine action [52, 53] (Fig. 1).

Anti-apoptosis is necessary for senescent cells to protect themselves from proapoptotic SASP molecules. To achieve this protection, senescent cells activate several anti-apoptotic pathways (SCAPs) [54], including B cell lymphoma family inhibitors (Bcl-2, Bcl-XL, Bcl-W), PI3K/Akt pathways, p53/p21Cip1/serpine pathways, HIF-1α, and HSP-90 [55, 56]. Therefore, senescent cells can accumulate through these anti-apoptotic pathways.

In addition, senescent cells are recognized and removed by the immune system to slow the progression of age-related diseases [57]. However, as aging progresses, the function of immune surveillance is largely diminished. As a result, senescent cells are not effectively phagocytosed or cleared timely [58], leading to the accumulation of senescent cells in the local joint which accelerates the progression of age-related diseases such as OA.

#### Autophagy-related signaling pathways

Autophagy is essential for maintaining the integrity and function of articular cartilage. However, the deficiency of autophagy results in cellular senescence [59–61]. In 2015, two consecutive reports appeared in *Science*, proposing that GATA4 is a key protein in the relationship between cell senescence and autophagy [62, 63]. More recently, it was found that METL3-mediated m6ATG7 modification regulates the autophagy-GATA4 axis to promote cell senescence and OA progression [64]. mTOR is a key regulator of autophagy and it is activated downstream of PI3 kinase and Akt kinase to inhibit autophagy. Studies have shown that mTOR is overexpressed in chondrocytes of OA patients and mouse models [65, 66]. Additionally, the PI3K/Akt/mTOR axis has been reported to regulate chondrocyte death in an OA rat model [67, 68]. Therefore, interfering the molecules in this signaling pathway may delay the progression of OA. Accumulating evidence also demonstrates that increased autophagy postpones cellular senescence by inhibiting the PI3K/Akt/mTOR signaling pathway. Beclin-1 promotes the formation of autophagosomes to induce autophagy. A study found that the expression of Beclin-1 inhibits the PI3K/Akt/mTOR signaling pathway thereby slowing the aging process of chondrocytes [69]. Furthermore, pituitary homeobox 1 (Pitx1) has been reported to be associated with cartilage degeneration [70]. Recently, a study confirmed that overexpression of Pitx1 increases the expression of SIRT1 and Beclin-1, inhibits the PI3K/Akt/mTOR signaling pathway, and increases autophagy levels thus preventing cellular senescence [71]. In contrast, targeted deletion of autophagy factor autophagy-related genes5 (ATG5), which is involved in the formation of complexes during autophagy, has been shown to promote age-related OA in mice and an increase in chondrocyte death was observed [72]. In view of the importance of autophagy in the pathogenesis of OA, studies have found that rapamycin, an mTOR inhibitor, activates autophagy to reduce the severity of osteoarthritis [73, 74].

Mitochondria are the “engines” of the cell, providing the cell with “fuel,” which is ATP, and participating in the energy metabolism of the cell [75]. Thus, mitochondrial homeostasis plays an important role in maintaining cellular function. Mitophagy is a key mechanism for maintaining mitochondrial homeostasis [76]. PTEN-induced putative kinase 1 (PINK1), a mitophagy-associated protein, has been found to be highly expressed in cartilage of OA patients and in mouse models of monosodium iodoacetate (MIA)-induced OA [77]. In addition, overexpression of parkin RBR E3 ubiquitin protein ligase (PRKN) reduces ROS production and chondrocyte apoptosis by removing dysfunctional mitochondria [78]. PINK1 and PRKN regulate mitophagy through the SIRT3-PINK1-PRKN signaling pathway, thereby delaying chondrocyte senescence [77, 78]. In addition, metformin has been found to act as an activator of the SIRT3-PINK1-PRKN signaling pathway and maintain chondrocyte homeostasis by regulating mitophagy [79]. These findings highlight the important role of the SIRT3-PINK1-PRKN signaling pathway in mitophagy and the maintenance of chondrocyte homeostasis, which provides a new direction for the treatment of OA.

### Skeletal cell senescence contributes to OA

**Fig. 2:**
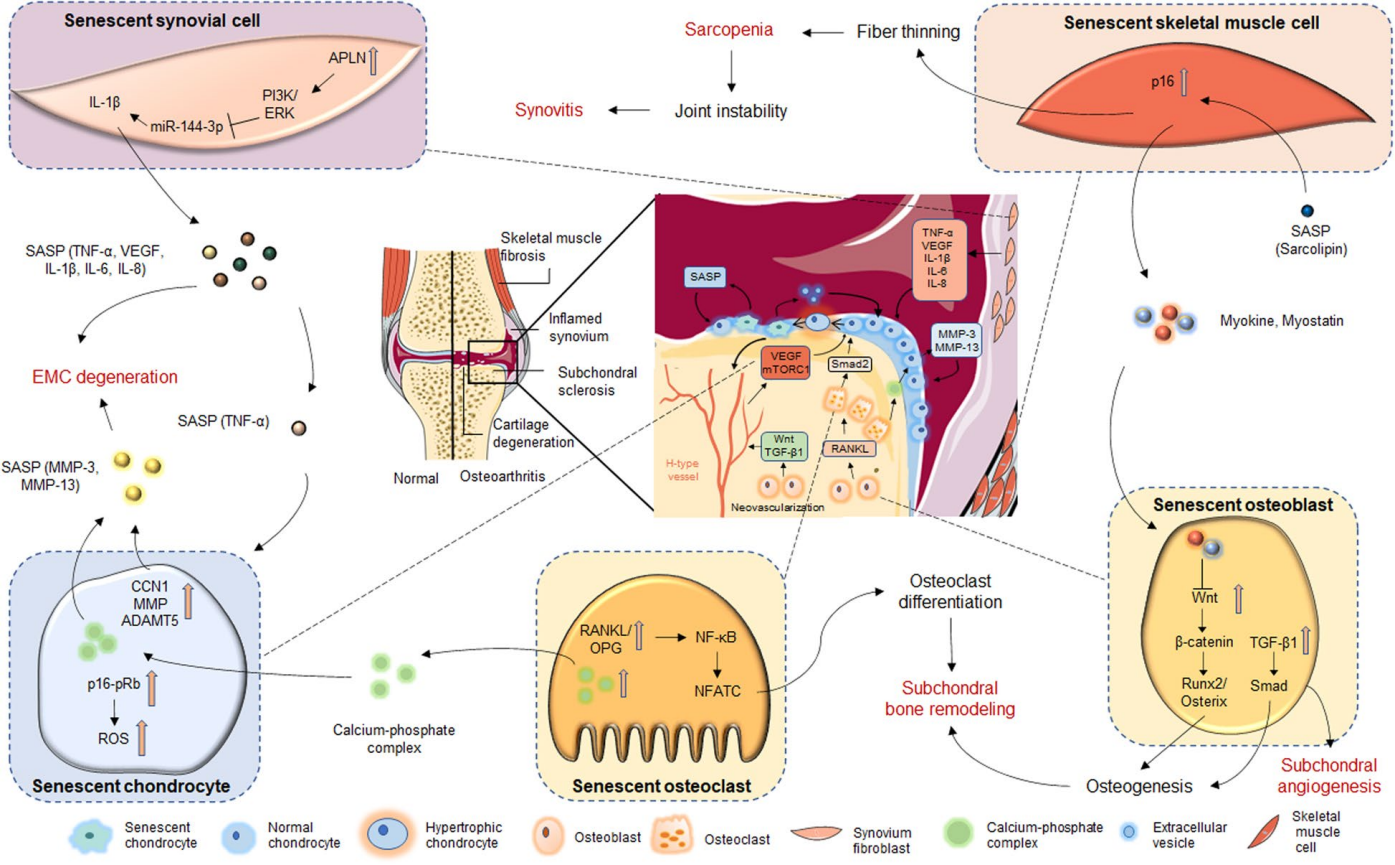
Multiple tissues within the knee contribute to OA progression. Senescent chondrocytes exert a pro-senescence effect on surrounding normal chondrocytes via SASP, and the senescent synovial cells can also act on normal chondrocytes via cytokines such as TNF-α, VEGF, and IL, leading to the cartilage degeneration. The process of subchondral bone degeneration in OA depends on osteoclast-mediated bone resorption and osteoblast-mediated bone formation. Osteoblasts act on osteoclasts via RANKL, which increases their activity accompanied by the secretion of large amounts of calcium-phosphate complex, thus promoting the secretion of MMP-3 and MMP-13 by the chondrocytes which causes cartilage degeneration. On the other hand, osteoblasts can induce subchondral angiogenesis via the TGF-β1/Smad signaling to indirectly induce senescent chondrocytes, which in turn, promote the secretion of more VEGF from the H-type vessel. All of these creating a vicious cycle of senescence and accelerating the development and progression of OA. SASP, senescence-associated secretory phenotype; TNF, tumor necrosis factor; TGFβ, transforming growth factor β; MMP, matrix metalloproteinase; RANKL, receptor activator of nuclear factor κB ligand; VEGF, vascular endothelial growth factor

**Fig. 3:**
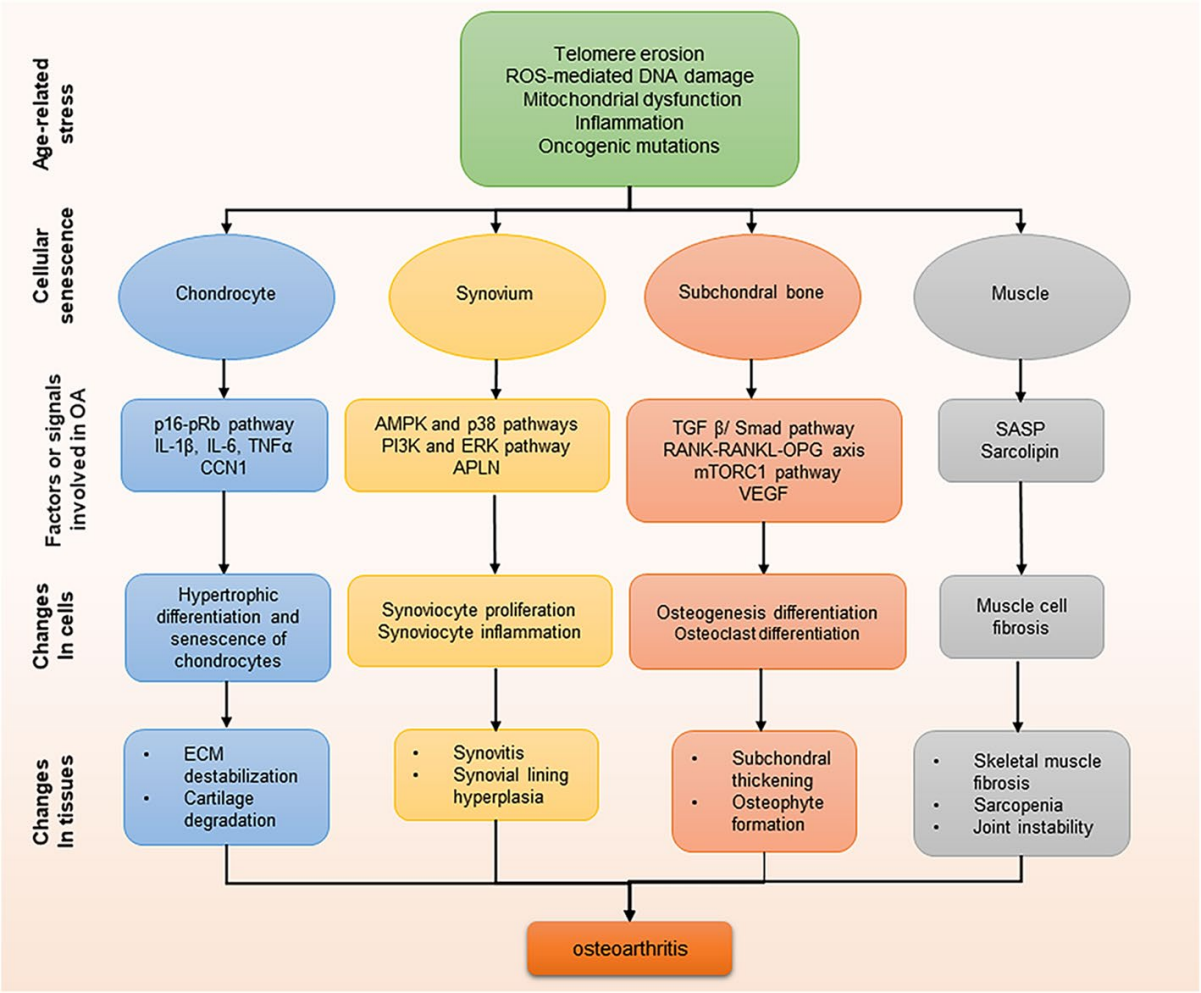
Associations between senescence and OA in different tissues. A variety of aging-related factors induce cellular senescence within joint tissues. These senescent cells in different tissues can lead to common changes in OA through the SASP or related signaling pathways. ROS, reactive oxygen species; IL, Interleukin; TNF, tumor necrosis factor; CCN1, Cellular communication network factor 1; ECM, extracellular matrix; AMPK, AMP-activated protein kinase; APLN, Adipokine apelin; PI3K, Phosphatidylinositol 3-kinase; RANKL, Receptor activator of nuclear factor κB ligand; VEGF, Vascular endothelial growth factor; TGFβ, transforming growth factor β; SASP, senescence-associated secretory phenotype

Increasing evidence has found that senescent cells exist in many tissues including cartilage, subchondral bone, and synovium of OA patients whom undergoing joint replacement. By studying a mouse strain (the p16-3MR transgenic mouse) that selectively develops senescent cells, Jeon et al. found that senescent cells accumulate in the articular cartilage, subchondral bone, and synovium in posttraumatic OA mouse model [80]. However, they did not further investigate the functional implications of these cells in OA development and progression. Senescent cells exhibit a typical SASP, which is observed in OA tissues and synovial fluid at high levels (Fig. 2). In this section, we review our current understanding of senescence in the skeletal cells and their cross-talk with each other within joints (Fig. 3).

#### Chondrocytes

Chondrocytes are unique cells in articular cartilage (AC) and are solely critical for the production and turnover of the extracellular matrix (ECM), which accounts for 95% of the AC [81]. The balance between the synthesis and degradation of matrix components in OA is an important factor in determining pathological progression.

Repeated mechanical trauma and oxidative stress are the two major contributing factors for establishing the chronic inflammatory microenvironment which triggers chondrocytes senescence [7]. Senescent chondrocytes acquire a secretory phenotype, releasing proinflammatory cytokines, vascular growth factors, and catabolic enzymes, which damage the stability of the ECM, thus initiating OA progression. This process is confirmed by an animal study showing that injection of senescent chondrocytes into the joints mimicked cartilage damage, similar to that observed in OA mice [82]. In contrast, removing senescent cells was demonstrated to reduce articular cartilage damage and pain in posttraumatic mouse models in another study [83]. These observations are largely explained by the fact that the acquisition of the SASP by senescent cells can change the joint microenvironment and thereby dictating the neighboring cells to undergo cell senescence. Additionally, p16 seems to be the most significant mediator and/or marker of senescent chondrocytes through the p16-pRb pathway, and downregulation of this gene has been shown to restore the normal function of OA chondrocytes [84].

On the other hand, senescent chondrocytes are unable to fold proteins properly. A misfolded protein, namely, the amyloid protein, can be detected in the cartilage of OA patients and has been shown to promote abnormal gene expression, mitochondrial dysfunction and cell death, thus affecting the energy metabolism of chondrocytes in patients with OA [85]. Mitochondria are the engines of cellular metabolism. Studies have shown that mitochondrial dysfunction was characterized by decreased expression of SIRT1 which was involved in mitochondrial biogenesis, and reduced expression of nuclear respiratory factors 1 and 2 (NRF1 and NRF2), which regulated antioxidant gene expression [86]. In addition, it was shown that the deletion of SIRT1 in cartilage aggravated the pathogenesis of OA [87]. Considering the results of the above studies, we conclude that chondrocyte senescence and OA are mutually reinforcing processes in OA initiation and progression.

SASP secreted by the senescent chondrocytes in OA play a negative role in regulating healthy neighboring chondrocytes via a paracrine mechanism [46, 88]. Recent studies have found that in addition to SASP, senescent cells are capable to secrete more extracellular vesicles when compared with the normal cells [89]. In addition, the secreted extracellular vesicles dictate the normal cell to undergo senescence via a paracrine manner and forming a vicious circle that might inhibit the cartilage formation [88]. In addition, a recent study has found that cell surface protein that mediates cellular communication such as cellular communication network factor 1 (CCN1) is increased in chondrocytes of OA patients. Inhibition of this protein could slow down the senescence of chondrocyte and reduce the production of MMPs and proinflammatory factors, thus protecting the articular cartilage [90]. These studies suggest that proteins mediating cellular communication may be considered as new targets for the intervention of OA.

##### Subchondral bone

In recent years, the role of subchondral bone in the pathogenesis of OA has gained increasing interest. Subchondral bone undergoes different pathological changes during the development of OA, in which subchondral bone wear occurs in the early stage, while the typical pathological changes in the late stage are subchondral bone sclerosis [91]. A recent study has suggested that the alteration of subchondral bone structure is the main cause of OA in animal models [92]. Fang et al. also found that early damage to subchondral bone (reduction of bone volume) occurs prior to cartilage degeneration and osteophyte formation, whereas in the late-stage of OA, this damage presents as subchondral bone sclerosis in mouse models, which is consistent with the typical pathology of patients with OA [91].

The pathological changes of subchondral bone in different stages of OA involve bone remodeling. Bone remodeling is highly relied on osteoclast-mediated bone absorption, osteoblast-mediated bone formation, and the interaction with osteocytes, which are embedded in the bone matrix (Table 2). In early-stage OA, the thickening of the calcified cartilaginous layer reduces the load on subchondral bone, and this reduction increases the ratio of receptor activator of nuclear factor κB ligand (RANKL) to osteoprotegerin (OPG) protein, thus promoting the formation of osteoclasts and enhancing bone resorption [95–97]. During subchondral bone degeneration, osteoclast activity is increased along with the secretion of a large amount of calcium-phosphate complex, thereby promoting the chondrocytes to secrete matrix metalloproteinase (MMP-3 and MMP-13) catabolic enzymes that cause cartilage degeneration [102]. Osteoclasts can also promote the hypertrophic differentiation of chondrocytes by targeting Smad2 [106], which further accelerated the degeneration of articular cartilage. Moreover, increasing evidence has suggested that bone remodeling of subchondral bone is associated with cellular senescence. In older animals, osteocytes and myeloid cells derived from trabecular and cortical bone tissue appear to senescence. In addition, the SASP of these cells is associated with age-related bone loss [80]. Furthermore, p16-positive senescent cells have been found in the subchondral bone marrow in the Anterior cruciate ligament transection (ACLT)-induced OA models [83]. Thus, senescent cells in the subchondral bone may cause physiological changes in the subchondral bone that affect bone remodeling thereby promoting OA progression.

**Table 2:** Role of various cells in the pathogenesis of OA

Cell types	Molecules or signals involved during OA	Effect	Refs
Chondrocytes	p16-pRb pathway	p16 induces senescence in OA chondrocytes via the p16-pRb pathway	[84]
	SASP (IL-1β, IL-6, TNF-α)	Senescent chondrocytes release SASP to destabilize the ECM, thereby initiating OA progression	[46, 88]
	CCN1	CCN1 signaling aggravates cartilage inflammation and matrix degradation.	[90]
	Amyloid protein	The amyloid protein promotes abnormal gene expression, mitochondrial dysfunction, and cell death in OA chondrocytes	[85]
Osteoblast	TGF β1; TGF β/ Smad pathway	Induce subchondral angiogenesis to indirectly affect chondrocyte	[93, 94]
	RANKL	Stimulate osteoclast differentiation and enhance bone resorption	[95–97]
	VEGF	Stimulate angiogenesis to indirectly affect chondrocyte	[98, 99]
	SOST	The lack of SOST aggravate OA by producing different degrees of apoptosis in the body to affect subchondral bone homeostasis	[100, 101]
Osteoclast	Calcium-phosphate complex	Promotes the secretion of MMP-3, MMP-13 by chondrocytes, leading to cartilage degeneration	[102]
	TGF β/Smad pathway	Promote the hypertrophic differentiation of chondrocyte	[93, 94]
Osteocyte	RANKL/OPG; RANK-RANKL-OPG system	Induce osteoclast differentiation and enhance bone resorption	[95–97]
	VEGF; mTORC1 pathway	Indirect regulation of chondrocytes by stimulating angiogenesis	[93]
Synovium fibroblasts	AMPK and p38 pathways; APLN; PI3K and ERK pathway	Knockdown of APLN expression could ameliorated changes in OA cartilage severity	[103]
Musculoskeletal cells	Sarcolipin	Sarcolipin secreted by senescent muscle cells promote skeletal muscle fibrosis and ultimately lead to sarcopenia, thereby accelerating the development of OA in terms of biomechanical mechanisms	[104]
	Myokine, myostatin; Wnt/β-catenin signaling	The muscle released myokine, myostatin inhibits osteogenic differentiation by suppressing Wnt/β-catenin signaling	[105]

*SASP* senescence-associated secretory phenotype, *IL* interleukin, *TNF* tumor necrosis factor, *TGFβ* transforming growth factor β, *CCN1* cellular communication network factor 1, *MMP* matrix metalloproteinase, *RANKL* receptor activator of nuclear factor κB ligand, *VEGF* vascular endothelial growth factor, *OPG* osteoprotegerin, *AMPK* AMP-activated protein kinase, *APLN* adipokine apelin, *PI3K* phosphatidylinositol 3-kinase

Furthermore, researchers have suggested that there is a positive feedback regulatory mechanism between subchondral H-type vessel formation and chondrocytes that is based on the mTORC1 pathway. In this mechanism, neovascularization of subchondral bone invades cartilage during cartilage degeneration, which stimulates the activation of the mTORC1 pathway by secreting more vascular endothelial growth factors (VEGFs), and the latter exacerbates the structural changes of the subchondral bone and drives OA progression rapidly. The same result was also observed in aged mice, and there was a prominent increase in H-type vessels in the subchondral bone of aged mice [98, 99]. These results suggested that the microenvironmental changes caused by senescent cells in the subchondral bone may affect the formation of H-type vessels, and H-type vessel formation in subchondral bone is closely related to the development of OA.

Bone remodeling is a process that coupling osteoclast-mediated bone resorption and osteoblast-mediated bone formation [107, 108]. Osteoblasts regulate cell mineralization by activating the Wnt signaling pathway, meanwhile, it can also respond to increased mechanical loading by reducing the secretion of sclerostin (SOST) [109, 110]. The lack of SOST aggravates OA by modulating the turnover and cell apoptosis of the subchondral bone, thus destructing the bone homeostasis [100, 101]. In addition, researchers have shown that osteoblasts can induce subchondral angiogenesis via the TGF-β1/Smad axis to indirectly affect the chondrocytes [93, 94]. Thus, inhibiting the damage to subchondral bone at the early stage via these pathways may be considered a method to treat OA.

##### Synovium

The synovium, which is composed of synovial fibroblasts and macrophages, is one of the main components of joints. Synovial cells secrete fluid to lubricate and nourish the joint. In clinical practice, patients with OA often suffer from synovial inflammation. Senescent cells have also been found in the synovium of OA patients [111]. In addition to senescent chondrocytes, the senescence of synovial fibroblasts is also thought to be the cause of OA. It has been demonstrated that more p16-positive senescent synovial fibroblasts are detected in synovial tissue samples from OA tissues compared with normal synovial tissue [112]. Furthermore, transplantation of senescent synovial fibroblasts into the knee of mice has been found to induce cartilage erosion and osteophyte formation [82], suggesting that senescent synovial cells play a key role in altering the intra-articular microenvironment thereby promoting OA pathogenesis. Mechanistically, senescent synoviocytes not only affect the quality of the synovium, but also increase the expression of IL-1β, IL-6, IL-8, TNF-α, and VEGF, which create an inflammatory microenvironment that favors cartilage degeneration [113]. TNF-α as proinflammatory mediator is crucial in regulating cartilage matrix degradation via upregulating the expression of matrix degradation enzymes such as MMPs and ADAMTS [114]. Even in the absence of an acute infection, the levels of inflammatory mediators in the joints of older OA patients usually increase with age [115]. .Although it is still unclear whether synovial changes are primary or secondary to OA, cross-talk between senescent skeletal cells and synovium is likely to be true. Synoviocytes induce cartilage destruction by secreting enzymes and inflammatory mediators that promote cartilage dissolution, thus aggravating synovial inflammation and forming a vicious cycle [116]. This cycle was exemplified in a recent study showing that adipokine apelin (APLN) in synovial fibroblasts of patients with OA regulates the activity of cartilage, synovium, bone, and various immune cells, and is related to the pathogenesis of OA [103]. Mechanistically, APLN inhibits the expression of miRNA-144-3p and stimulates the expression of IL-1β by activating the PI3K and ERK pathways [103]. Thus, downregulating the expression of APLN may ameliorate changes in OA cartilage severity.

##### Musculoskeletal cells

Decreasing activity and muscle wasting would be caused by OA pain, and we have recently demonstrated that knee muscle atrophy is a risk factor for development of knee osteoarthritis [117]. Adult muscle stem cells in skeletal muscle are called satellite cells which are mainly responsible for muscle repair and regeneration in injured and aging muscles. However, the repair and regenerative function of satellite cells decreases with aging [118]. It has been found that when injured, these cells accelerate into senescence losing their repair and regenerative functions [17]. A recent study has shown that the implantation of senescent cells into the healthy skeletal muscle resulted in an increase of p16-positive muscle cells, along with the thinning of muscle fiber [119]. Factors in SASP such as sarcolipin have also been shown to promote skeletal muscle fibrosis and ultimately lead to sarcopenia [104]. Sarcopenia due to senescent skeletal muscle cells may also be involved in the development of OA from a biomechanical mechanism, e.g., causing joint instability or secondary synovitis. Furthermore, the muscle-released myokine, myostatin, is demonstrated to inhibit osteogenic differentiation by suppressing the Wnt/β-catenin signaling [105]. These findings together suggest that the senescence of musculoskeletal cells via cross-talk with synovium fibroblasts, chondrocytes, and osteoblast, is fundamental for OA initiation and progression.

### Targeted therapies for age-related OA

**Fig. 4:**
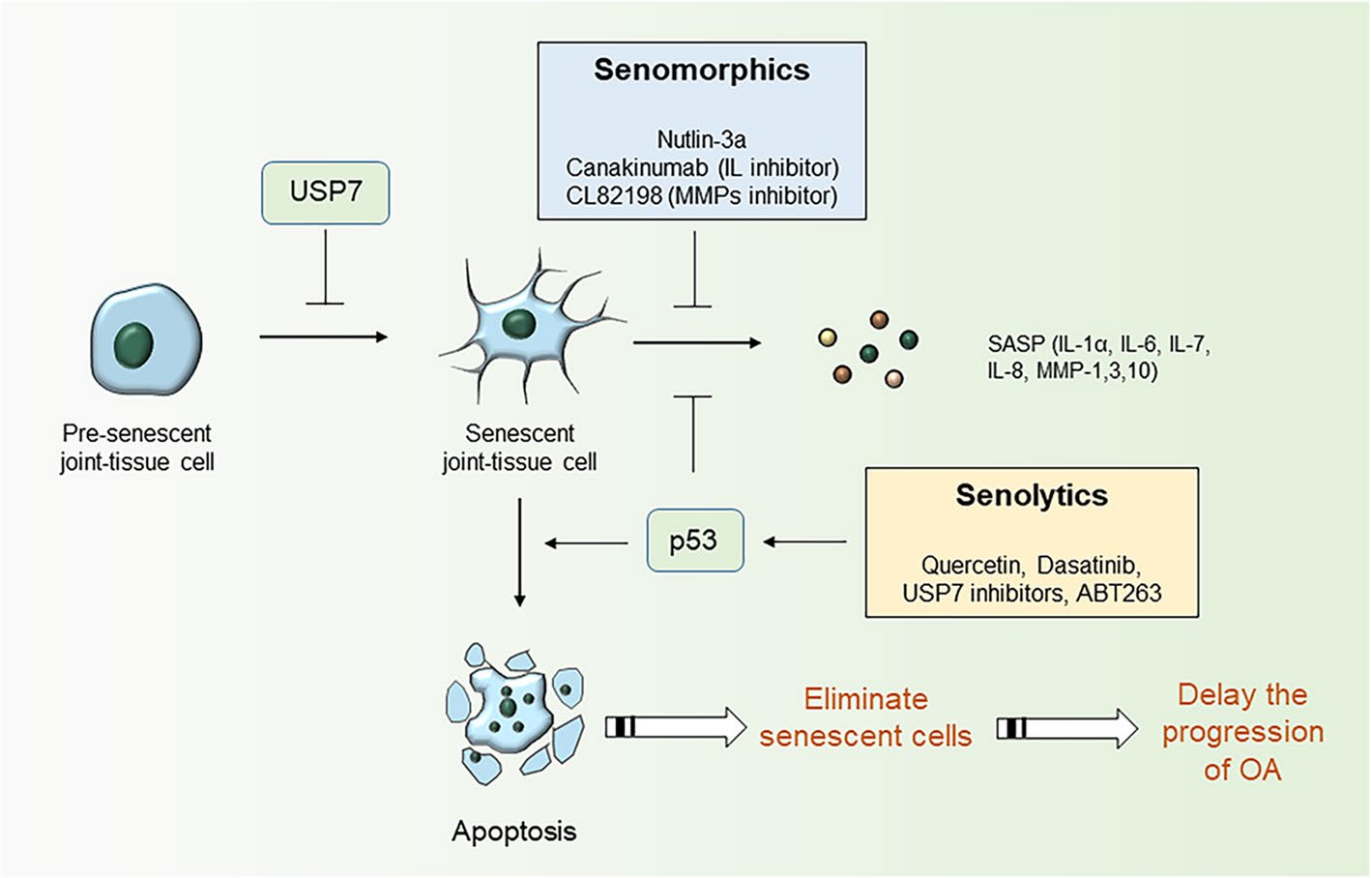
Targeted therapies for senescent cells in joint tissues. Senolytics (Quercetin, Dasatinib, USP7 inhibitors, ABT263) by inducing the apoptosis of senescent cells and Senomorphics (Nutlin-3a, Canakinumab, CL82198) by blocking the paracrine secretion of senescent secretory phenotype (SASP) to delay the effect of senescent cells on the development of OA. Senolytics induce apoptosis in senescent cells by restoring the activity of p53. Senomorphics inhibit the secretion of SASP to attenuate the pro-senescence effect of senescent cells on surrounding cells

As mentioned above, senescent cells in joints contribute to the senescence of neighboring cells through the SASP in the form of paracrine signaling. Senescent cells further promote the accumulation of senescent cells through the activation of SCAPs, thereby exacerbating the development of OA. Therefore, local elimination of senescent cells and/or blockade of SASP acquisition are considered promising therapeutic strategies for OA. A summary utilizing these principles to delay the progression of OA can be found in Fig. 4 and Table 3.

**Table 3:** Targeted therapies for OA

Categories of drugs	Compound	Effects in OA	Refs
Senolytics	Quercetin and Dasatinib	Selectively induce apoptosis in senescent cells while not affecting proliferating cells	[120–122]
	USP7 inhibitors	Activate the apoptotic program after the stabilization of p53 to kill senescent cells	[123]
	ABT263 (Navitoclax)	Eliminate senescent cells, reduce the expression of inflammatory cytokines and promote the maintenance of chondrogenic phenotypes in vitro	[111]
Senomorphics	Nutlin-3a	Increase p53 levels and attenuate the secretory phenotype of senescent cells	[124, 125]
	Canakinumab	Inhibition of IL-1β expression delays the progression of OA	[126]
	CL82198	Reduce the severity of OA by inhibiting chondrocyte death	[127]

Recently, according to the inflammatory mechanisms, cell aging, cartilage metabolism, subchondral bone remodeling, and peripheral pain pathways, a series of therapies have been developed against OA [128, 129]. Senolytics and Senomorphics are promising therapeutic agents that slow the progression of OA by targeting its pathogenesis [130]. Senolytics induces the apoptosis of senescent cells and Senomorphics blocks the paracrine secretion characteristic of the SASP to eliminate or delay the adverse effects of senescent cells in the development of OA [131]. Senolytics is a drug selectively eliminating senescent cells. Dasatinib, the first generation of Senolytics, which is a chemotherapy drug used to treat leukemia, and later, it was developed as a therapeutic strategy eliminating senescent cells. Research has shown that quercetin and Dasatinib selectively induce the apoptosis of senescent cells but do not affect proliferating cells [120]. In another study, transplantation of senescent cells into young mice resulted in physical dysfunction in the mice. However, treating these mice with quercetin and dasatinib mitigated the harmful effects caused by senescent cell transplantation [121, 122]. Mouse double minute 2 (MDM2) is one of the most important inhibitors of p53. When these two proteins are combined, they degrade the p53 protein and reduce its activity. A study has shown that ubiquitin-specific peptidase 7 (USP7) was a novel target of Senolytics, because it isolated p53 from MDM2, thereby inhibiting the MDM2 interaction with p53 in mice, and selectively eliminating senescent cells by restoring p53 activity. Furthermore, this study implicated that injection of USP7 inhibitors into the joint of OA rats inhibited cartilage degeneration, thereby slowing the progression of OA [123]. Recently, a study evaluated the ability of the senolytic drug ABT263 (Navitoclax) to clear senescent cells. Chondrocytes were obtained from OA patients for use in experiments performed in vitro and in a DMM rat model for in vivo experiments to evaluate the effect of ABT263. The results showed that ABT263 reduced the expression of inflammatory cytokines in OA cartilage cultures and promoted cartilage matrix aggregation by inducing the apoptosis of senescent cells. Intra-articular injection of ABT263 attenuated bony lesions in cartilage and subchondral bone of rats with DMM-induced OA [111]. Considering the correlation between SASP factor expression and the development of OA, inhibition of SASP molecule secretion is a promising therapeutic approach. One study has confirmed that Nutlin-3a, a Senomorphics, increases p53 expression and attenuates the SASP. Therefore, Nutlin-3a prevents OA development and cartilage destruction by inhibiting MDM2 expression and activating the apoptotic program to kill senescent cells after p53 stabilization [124, 125]. Another study found that in an experimental group receiving canakinumab, an inhibitor of IL-1β, the incidence of knee and hip replacements was lower than that in the placebo-treated control group [126]. Additionally, some researchers have used the MMP13 selective inhibitor CL82198 to treat meniscal-ligamentous injury-induced OA mouse models. They found that CL82198 reduced the severity of OA by inhibiting chondrocyte death [127]. These findings suggested that Senomorphics also delay the progression of OA.

In conclusion, the removal of senescent cells and/or suppression of the SASP subsequently slow the progression of OA. Therefore, targeting senescent cells and their SASP may provide a novel strategy for the treatment of age-related diseases such as OA.

### Experimental animal models for the research of OA and aging

Experimental animal models are essential tools for exploring the mechanism of OA development and progression. The mechanisms between OA and cellular senescence are complex. Therefore, the establishment of an ideal animal model is particularly important for the study of OA pathogenesis and/or evaluation of the curative effect. A summary of the animal models currently used for aging and OA studies are shown in Table 4 and Table 5.

**Table 4:** The advantages and disadvantages of experimental animal models for OA and aging

Exp. animal models	Advantages	Disadvantages
Spontaneous model	Mostly similar to the characteristics of human aging, omitting surgery, or drug delivery steps	Long feeding period, poor health, high mortality, and large individual differences
Drug-induced model	Little trauma, no traumatic synovitis interference, simple operation	Cannot accurately simulate the chronic changes of human osteoarthritis
Surgical induction model	Short period of establishing model time, high success rate, and high repeatability	Complicated operation, large trauma; hemorrhage and traumatic synovitis easily affect the biochemical metabolism of cartilage and synovium in the early stage of OA
Transgenic model	Accurate modification to facilitate the study of mechanism	The model establishing process is long and costly.

**Table 5:** Experimental animal models for the research of OA and aging

Exp. animal models for OA	Refs	Exp. animal models for aging	Refs
Spontaneous model of OA		Transgenic mouse strains	
STR/ort mice	[132]	p16^LUC^ knock-in mice	[133]
SAMP8 mice	[134]	Cdkn2a^luc/luc^ mice	[134]
Surgically induced OA models		p16-3MR transgenic mice	[83]
ACLT	[135]	SAMP8 mice	[136]
DMM	[137]	D-Galactose-induced model	[138]
Transgenic and inbred mouse strains		Slc25a46- knockout mice	[139]
ADAMTS5-knockout mice	[140]		
SIRT1-knockout mice	[87]		
JNK-knockout mice	[36]		
AMPK α1-knockout mice	[141]		
Pitx1-knockout mice	[71]		
Chemically induced models			
Injections of MIA	[77, 142, 143]		

*SAMP* senescence-accelerated mouse-prone, *ACLT* anterior cruciate ligament transection, *DMM* destabilization of the medial meniscus, *SIRT1* Sirtuin 1, *JNK* Jun N-terminal kinases, *AMPK* AMP-activated protein kinase, *Pitx1* pituitary homeobox 1, *MIA*, monosodium iodoacetate

#### Spontaneous model of OA

The spontaneous model does not need intervention and is less subject to external interference. It avoids the influence caused by operation error as much as possible and is closer to the OA process of humans. However, due to the slow progress, the application in the research is limited. There is a spontaneous model of OA, STR/ort mice, which spontaneously develop OA in early life and show many characteristics of human OA [132]. Furthermore, Malaise et al. firstly showed that senescence-accelerated mouse-prone (SAMP8) mice developed OA spontaneously [134]. The use of these spontaneous OA mice can avoid interference from external factors, and thus, the pathology of age-related OA can be more accurately mimicked.

#### Inductive model and transgenic animal model of OA

Inductive models of OA are established through surgical and chemical interventions. Surgically induced OA models were usually established using ACLT or medial meniscus instability (DMM). These methods were firstly used in mice by the Kamekura group [135]. Mechanical instability-induced models establish models with slower OA progression, which is comparable to the development of human OA. The DMM model simulates clinical meniscus or ligament injury, which is a known risk factor for the onset of human OA. This model has become the gold standard for OA research [137].

With the development of transgenic technology, transgenic and inbred mouse strains are particularly useful models for studying the molecular mechanisms of OA development. The DMM model has been established with transgenic mice to evaluate the effect of specific genes on the development and progression of OA. Glasson et al. first reported that single-gene deletions can reduce cartilage destruction in an animal model of OA. They demonstrated that the severity of cartilage damage in ADAMTS5-knockout mice was significantly lower than that in wild-type mice [140]. Recently, using the same approach, Mao et al. found that the deletion of SIRT1 in cartilage exacerbated the pathogenesis of OA by activating p53/p21-mediated cellular senescence and increasing SASP-related secretion levels [97]. .Furthermore, Loeser et al. found that the deletion of JNK1 or JNK2 can aggravate the development of age-related OA by increasing the number of senescent cells in JNK-knockout mice [36]. In a recent study, AMP-activated protein kinase (AMPK) α1-knockout mice were used to study the effects of metformin on the progression of OA [106]. The results showed that the DMM-induced OA phenotype was accelerated in AMPK α1-KO mice. Metformin has a protective effect on cartilage and can reduce the progression of OA, but metformin lost its protective effect in the AMPK α1-KO mice, which suggests that the cartilage protection of metformin is mediated by AMPK signaling [106]. Furthermore, a recent study found that activation of AMPK resulted in decreased expression of the SASP in senescent cells [144]. Therefore, we hypothesize that the protective effect of metformin on cartilage in OA patients may be due to the activation of the AMPK pathway and the reduction of the secretion in SASP-related secretions from senescent chondrocytes. AMPK α1-KO mice may also be useful in the study of aging and OA. Pitx1 plays a role in regulating lower limb development [141]. In consideration of the characteristics of uneven cartilage degeneration in OA, Zhao et al. first proposed a self-controlled model to investigate the effects of Pitx1 on chondrocytes of OA cartilage. The results revealed that downregulation of Pitx1 was an important cause of cartilage degeneration and decreased chondrocyte function and chondrocyte senescence in the medial tibial plateau of OA patients [71]. This study found that downregulation of Pitx1 caused chondrocyte senescence in the OA patients; thus, Pitx1-knockout mice may also be a suitable model for aging and OA studies.

Establishing chemically induced models involves injections of inflammatory agents such as immunotoxins, collagenase, papain, or MIA into the knee [142, 145, 146]. The MIA model has become a standard for modeling OA models in animals. Intra-articular injection of MIA destroys glycolysis in chondrocytes, leading to chondrocyte death, neovascularization, subchondral osteonecrosis, and collapse, as well as inflammation that mimics that of OA [142, 143]. However, some studies have reported that abnormal glycolysis can lead to cellular senescence [147]. Therefore, intra-articular injection of MIA may aggravate the development of OA by accelerating cellular senescence in the joint.

#### Experimental animal models of aging

Monitoring aging in vivo remains challenging in experiments. Burd et al. described a luciferase knock-in mouse (p16^LUC^) that can specifically reveal the expression of p16 and therefore can be used for aging-related studies [133]. To test whether senescent cells aggravate the development of OA. Malaise et al. established Cdkn2a^+/luc[+/luc]^ and Cdkn2a^luc/luc^ transgenic mice, in which the Cdkn2a^luc/luc^ mice lack p16 expression. They found that the loss of p16 expression after OA induction had a significant protective effect on OA-driven cartilage degradation [134]. Furthermore, another study demonstrated that p16-positive cells play deleterious roles in ACLT-induced OA joints through p16-3MR transgenic mice, which specifically track and remove senescent cells [83]. In addition, the mice mentioned above and the D-Galactose-induced accelerated aging models can be used in the study of age-related diseases or anti-aging therapeutic intervention studies [136, 138]. Recently, a mouse model with the Slc25a46 gene knocked out was developed using CRISPR/Cas9 gene editing to mimic some of typical aging phenotypes in humans, and it was an effective animal model for pathological study of segmental aging based on mitochondrial theory [139]. The Slc25a46-KO mouse is characterized by a life span of no more than 2 months, skeletal muscle dysfunction, and reduced exercise capacity; thus, this animal model can be used in the study OA because it simulates the aging state of humans realistically.

### The future direction of aged-related OA research

**Fig. 5:**
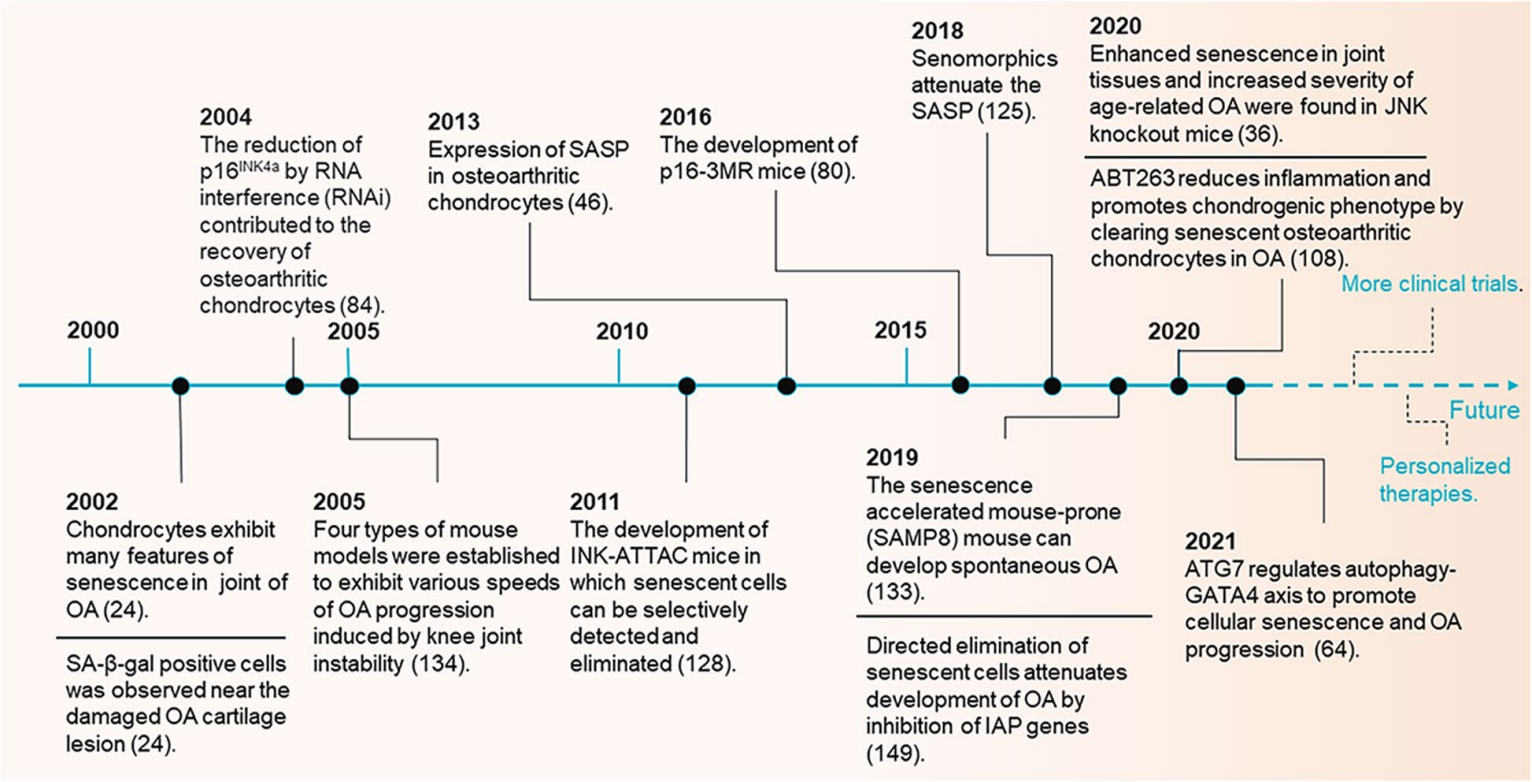
Timeline of age-related OA research over the past 20 years. Key discoveries in OA from the perspective of cellular senescence are highlighted. Since 2002, the mechanism of chondrocyte aging on the pathogenesis of OA has been first reported, and by 2021, a variety of anti-aging drugs have been discovered to treat OA. The continuous update of treatment methods mainly depends on the continuous in-depth understanding of the pathogenesis of OA by researchers

Research on age-related OA over the past 20 years has transitioned from identifying phenotypes to studying the genetic and epigenetic signaling behind these phenotypes. Cellular senescence and OA are interactive processes involving a complex network of intracellular signaling pathways. In Fig. 5, we recapitulate a few key processes that have been discovered during the past 20 years, which contribute to the understanding of how cellular senescence is involved in the study of OA.

To understand the underlying mechanism between cellular senescence and OA, many animal models have been introduced. Subsequently, transgenic mice have also been studied in the field of aging-related OA. The development of transgenic mouse models that selectively eliminate senescent cells has confirmed that senescent cells play important roles in the development of OA. For example, the p16-3MR transgenic mice described above carry the p16INK4a (Cdkn2a) promoter, which drives the expression of a fusion protein containing the structural domains of synthetic luciferase and fluorescent protein [83, 133], thereby allowing tracking of p16-positive cells.

Using appropriate animal models of aging and OA might provide better evidence to improve our understanding of the underlying mechanisms of age-related OA. Furthermore, studies are required in which OA can be considered a “whole organ” disease and show the cross-talk between senescent skeletal cells and macrophage, which is of great importance. Using these models, we can prevent, delay, or reverse cellular senescence within the joints, possibly, by using Senolytics and Senomorphics to halt the development of OA [120, 148, 149].

## Conclusion

Over time, cellular senescence in joint tissue is inevitable. The evidence suggests that senescent cells may be involved in the onset and progression of OA through the SASP and/or altering the intra-articular joint environment. Senescence is closely associated with OA, and the pathogenesis of age-related OA involves all components of the joint, including articular cartilage, subchondral bone, synovium, and muscles. Senescent skeletal cells may influence the development of OA through cross-talk with synovial cells. In addition, the interaction of senescent cells in joint tissue might disrupt intra-articular homeostasis, leading to exacerbation of OA. Therefore, it is suggested that the cross-talk between senescent skeletal cells and synovial cells within joints is critical for initiating OA. However, further research is needed to determine the precise mechanism(s) by which senescence induces acquisition of these specific phenotypes. If we can identify the cells that are the major risk factors for OA, then administration of a specific drug eliminating these senescent cells may become a new method of treating OA, with the potential to prevent and treat it in the near future. Recently, several therapeutic drugs including Senolytics and Senomorphics have been developed and tested in a panel of cells or animals. More evidence has shown that targeting senescent cells will shed light on the treatment of age-related chronic diseases such as OA.

## Data Availability

Not applicable.

## Abbreviations

AC: Articular cartilage
ATG: Autophagy-related gene
APLN: Adipokine apelin
ACLT: Anterior cruciate ligament transection
AMPK: AMP-activated protein kinase
COL10A1: Collagen type X
COL2A1: Collagen type II
CCN1: Cellular communication network factor 1
DMM: Destabilization of the medial meniscus
ECM: Extracellular matrix
GM-CSF: Granulocyte macrophage-colony stimulating factor
HSV-TK: Herpes simplex virus 1 thymidine kinase
IL-1β: Interleukin-1β
IL-6: Interleukin-6
JNK: Jun N-terminal kinase
MSCs: Mesenchymal stem cells
MAPK: Mitogen-activated protein kinase
MDM2: Mouse Double Minute 2
MIA: Monosodium iodoacetate
MP: Microparticle
mTOR: Mechanistic target of rapamycin kinase
NRF: Nuclear respiratory factors
NF-κB: Nuclear factor κB
OA: Osteoarthritis
OPG: Osteoprotegerin
PTOA: Posttraumatic osteoarthritis
PI3K: Phosphatidylinositol 3-kinase
Pitx1: Pituitary homeobox 1
PINK1: PTEN-induced putative kinase 1
PRKN: Parkin RBR E3 ubiquitin protein ligase
ROS: Reactive oxygen species
RTL: Relative telomere length
RANKL: Receptor activator of nuclear factor κB ligand
SASP: Senescence-associated secretory phenotype
SA-β-gal: Senescence-associated beta-galactosidase
SAMP: Senescence-accelerated mouse-prone
SCAPs: Senescent cells anti-apoptotic pathways
SIRT1: Sirtuin 1
SOX9: SRY-BOX transcription factor 9
SOD2: Superoxide dismutase 2
SOST: Sclerostin
TGFβ: Transforming growth factor β
USP7: Ubiquitin-specific peptidase 7
VEGF: Vascular endothelial growth factor
